# Prevalence of overweight, obesity, abdominal obesity and obesity-related risk factors in southern China

**DOI:** 10.1371/journal.pone.0183934

**Published:** 2017-09-14

**Authors:** Lihua Hu, Xiao Huang, Chunjiao You, Juxiang Li, Kui Hong, Ping Li, Yanqing Wu, Qinhua Wu, Zengwu Wang, Runlin Gao, Huihui Bao, Xiaoshu Cheng

**Affiliations:** 1 Department of Cardiovascular Medicine, the Second Affiliated Hospital of Nanchang University, Nanchang of Jiangxi, China; 2 Division of Prevention and Community Health, National Center for Cardiovascular Disease, Fuwai Hospital, Peking Union Medical College & Chinese Academy of Medical Sciences, Beijing, China; 3 Fuwai Hospital, Peking Union Medical College & Chinese Academy of Medical Sciences, Beijing, China; Shanghai Institute of Hypertension, CHINA

## Abstract

**Objectives:**

The purpose of this study is to assess the prevalence of overweight/obesity, abdominal obesity and obesity-related risk factors in southern China.

**Methods:**

A cross-sectional survey of 15,364 participants aged 15 years and older was conducted from November 2013 to August 2014 in Jiangxi Province, China, using questionnaire forms and physical measurements. The physical measurements included body height, weight, waist circumference (WC), body fat percentage (BFP) and visceral adipose index (VAI). Multivariate logistic regression analysis was performed to evaluate the risk factors for overweight/obesity and abdominal obesity.

**Results:**

The prevalence of overweight was 25.8% (25.9% in males and 25.7% in females), while that of obesity was 7.9% (8.4% in males and 7.6% in females). The prevalence of abdominal obesity was 10.2% (8.6% in males and 11.3% in females). The prevalence of overweight/obesity was 37.1% in urban residents and 30.2% in rural residents, and this difference was significant (*P* < 0.001). Urban residents had a significantly higher prevalence of abdominal obesity than rural residents (11.6% vs 8.7%, *P* < 0.001). Among the participants with an underweight/normal body mass index (BMI), 1.3% still had abdominal obesity, 16.1% had a high BFP and 1.0% had a high VAI. Moreover, among obese participants, 9.7% had a low /normal WC, 0.8% had a normal BFP and 15.9% had a normal VAI. Meanwhile, the partial correlation analysis indicated that the correlation coefficients between VAI and BMI, VAI and WC, and BMI and WC were 0.700, 0.666, and 0.721, respectively. A multivariate logistic regression analysis indicated that being female and having a high BFP and a high VAI were significantly associated with an increased risk of overweight/obesity and abdominal obesity. In addition, living in an urban area and older age correlated with overweight/obesity.

**Conclusion:**

This study revealed that obesity and abdominal obesity, which differed by gender and age, are epidemic in southern China. Moreover, there was a very high, significant, positive correlation between WC, BMI and VAI. However, further studies are needed to explore which indicator of body fat could be used as the best marker to indirectly reflect cardiometabolic risk.

## Introduction

Obesity has become a growing global public health problem, owing to its high prevalence and substantial morbidity and mortality. Obesity and abdominal obesity are associated with an increased risk of multiple chronic diseases, including diabetes, cardiovascular disease (CVD), hypercholesterolemia, asthma and cancer [1,2]. It has been reported that there are approximately 937 million obese adults and 396 million overweight adults worldwide [3]. As mentioned above, obesity has rapidly been established as a public health problem. Hence, in 2013, the American Medical Association called for physicians to focus on obesity. Over the past decade, China has seen rapid economic growth that has led to changes in dietary and physical activity patterns and an increase in life expectancy, which, in turn, has led to an increase in obesity prevalence, especially in large cities [4,5]. Previous studies have also showed that the prevalence of obesity increased from 4.0% in 1993 to 10.7% in 2009 and an increase in the overweight prevalence by 67% from 9.4% to 15.7% was also observed over this time period [5]. Although many studies have focused on overweight/obesity and abdominal obesity, there are still noticeable ethical and geographical differences that exist. However, there are still no large-scale surveys published on the obesity prevalence in southern China, especially in Jiangxi Province.

Moreover, there are many indicators that inflect body fat distribution, including body mass index (BMI), waist circumference (WC), visceral adipose index (VAI), body fat percentage (BFP) and waist-to-height ratio (WtHR). Obesity, especially abdominal obesity, is associated with increased, cardiovascular, cancer, and all-cause mortality [6]. Although BMI is now considered as a clinical or epidemiological tool for the evaluation of cardiovascular risk in both primary and secondary prevention, some studies still suggest that BMI is not a good predictor of mortality risk [7,8]. Obesity-related comorbidities were found to be more closely associated with abdominal adiposity and visceral fat depots than with the amount of total body fat [9]. VAI in the general population can be used a marker that indirectly reflects cardiometabolic risk [10,11]. Despite this, it is still controversial [10–12]. Thus, our study aims to describe the up-to-date prevalence and correlates of overweight/obesity and abdominal obesity, which may provide effective guidance on intervention strategies for obesity. Additionally, the study shows the associations between WC, BMI, VAI and BFP, to suggest a better epidemiological tool for the evaluation of cardiovascular risk.

## Methods

### Ethics statement

Ethical approval was obtained from the ethics review boards of the Second Affiliated Hospital of Nanchang University and the Fuwai Cardiovascular Hospital (Beijing, China). Written informed consent was obtained from each participant and the guardians on behalf of the minors/children aged 15–18 years old who were enrolled in the study. If the guardians were unable to write, then fingerprinting was used. The ethics committee approved the procedure.

### Study design and subjects

Four cities in urban areas and four counties in rural areas were selected using the probability-proportional-to-size method, in which two districts or two townships were selected. Then, three communities or villages were chosen within each district and township, respectively, using the simple random sampling (SRS) method. A given number of participants from each of the 14 gender/age strata (male/female and participants aged 15–24, 25–34, 35–44, 45–54, 55–64, 65–74, and ≥ 75 years) was chosen, also using the SRS method according to the national demographic composition, from communities or villages using lists compiled from the local government registers of households [13,14]. The design effect was also considered while estimating the sample size. Assuming a design effect of 2.5 and a prevalence of hypertension of 17.7% among the population aged 15 years and older, 15,200 participants were estimated for the analysis to ensure that the average lengths of the 95% confidence intervals for the prevalence in the entire population and in the subpopulation defined by age and gender were less than 0.4% and 1.8%, respectively [13,14].In total, 15,364 residents participated in the study from November 2013 to August 2014, and 400 participants with missing information on sex, age, weight, height, and/or waist circumference were excluded; therefore 14,964 participants were included in the analysis.

### Data collection procedure

Participants were required to complete a questionnaire that was developed by the national coordinating center, at Fuwai Cardiovascular Hospital; this questionnaire was conducted through face-to-face interviews by trained staff and included physical measurements using standardized procedures. Data obtained from the questionnaire included personal basic information (such as age, gender, marital status, area, education), and behavioral characteristics (such as smoking habit, and alcohol consumption). The anthropometric examinations included body height, weight, WC, BFP and VAI. All of the investigators were medical students who were systematically trained. In addition, standard protocols and instruments were used. The certification requirements for data collection were strict, and a quality assurance program was conducted.

### Anthropometric measurements

Body weight without heavy clothing, as well as BFP, and VAI were measured using an Omron body fat and weight measurement device (V- BODY HBF-371, Omron, Kyoto, Japan). Height was measured without shoes using a standard right-angle device and a fixed measurement tape (to the nearest 0.5 cm). Waist circumference was measured (to the nearest 0.5 cm) by putting the measuring tape at the midpoint between the lower margin of the last rib and the top of the hip bone (at the level of umbilicus) at the end of expiration. All measurements were taken twice and the average of the 2 values was adopted.

### Definitions

BMI was calculated as the weight in kilograms divided by height in meters squared (kg/m^2^). Overweight and obesity were defined as a BMI of 24–27.9 kg/m^2^ and a BMI ≥ 28 kg/m^2^, respectively [14,15]; BMI ≥ 24 kg/m^2^ was defined as an elevated BMI. WC was divided into abdominal overweight (85–95 cm in males and 80–90 cm in females) and abdominal obesity groups (WC ≥ 95 cm in males and ≥ 90 cm in females) [14,15]. WC ≥ 85 cm in males and ≥ 80 cm in females were defined as elevated WC. VAI was categorized into three groups as standard (1–9), slightly high (10–14) and high (15–30) [14,16]. BFP was categorized into four groups: thin (< 10% for males and < 20% for females), standard (10–19% for males and 20–29% for females), slightly high (20–25% for males and 30–35% for females), and high (≥ 25% for males and ≥ 35% for females) [14,17]. Cigarette smokers were defined as having smoked at least one cigarette per day for 6 months or more [14]. Alcohol use was defined as drinking alcohol at least one time per week during the previous year [14].

### Statistical analysis

All data were established using EpiData version 3.02 software. After alignment correction, a statistical analysis was performed using the Statistical Package for Social Science software 17.0 (SPSS, IL, USA). Continuous variables are presented as the mean ± standard deviation or the median (IQR), as appropriate, and are compared using the *t* test or the Mann–Whitney *U* test, depending on whether the quantitative data were consistent with a normal distribution. Categorical variables are expressed as percentages and were analyzed using the chi-square test or Fisher’s exact test as appropriate. Partial correlation analysis was used to evaluate the association between WC, BMI, VAI and BFP. Multivariate logistic regression analysis was carried out to evaluate the risk factors for elevated BMI and abdominal obesity as the dependent variables. A value of p < 0.05 was considered statistically significant.

## Results

As shown in S1 Table, a total of 14,964 participants from 15,364 initial participants were included in the analysis, including 6,127 males and 8,837 females, with a mean age of 56 years and a median age of 53 years. The response rate was 97.4%. The proportion of rural and urban residents was similar within different sexes. Compared with females, males showed higher values for age, BMI, WC and VAI but not for BFP. 400 participants were excluded because of missing information on sex, age, weight, height, and/or waist circumference. The majority of nonresponders were young, and their lack of response was likely due to their busy work schedules.

### Prevalence of overweight/obesity and abdominal obesity

Table 1 shows that the overall prevalence of overweight, obesity and abdominal obesity was 25.8%, 7.9% and 10.2%, respectively. In males, 25.9% were overweight and 8.4% were obese. Likewise, in females, 25.7% was overweight and 7.6% was obese. There was a non-significant tendency regarding the prevalence of overweight and obesity in both genders. Females were more likely than males to be placed in the categories of abdominal overweight and abdominal obesity, with a prevalence of 29.0% and 11.3%, respectively. Moreover, the prevalence of elevated WC was statistically significantly associated with gender (*P* < 0.001).

**Table 1:** Characteristics of participants stratified by sex.

variables	Total (n = 14,964)	Male (n = 6,127)	Female (n = 8,837)	*p*-value
Gender (%)		40.9	59.1	
Age (years) M (P25~P75)	54 (41~67)	56.0 (40~68)	53.0 (41.0~66.0)	0.009
Urban *N* (%)	7645 (51.1)	3200 (52.2)	4445 (50.3)	0.020
Rural *N* (%)	7319 (48.9)	2927 (47.8)	4392 (49.7)	
Current smokers *N* (%)	2705 (18.1)	2542 (41.5)	163 (1.8)	<0.001
Current drinkers *N* (%)	3569 (23.9)	2408 (39.3)	1161 (13.1)	<0.001
BMI (kg/m^2^) M (P25~P75)	22.5 (20.3~25.0)	22.6 (20.3~25.1)	22.4 (20.3~24.9)	0.195
Under/Normal *N* (%)	9916 (66.3)	4022 (65.7)	5894 (66.7)	0.143
Overweight *N* (%)	3862 (25.8)	1589 (25.9)	2273 (25.7)	
Obesity *N* (%)	1186 (7.9)	516 (8.4)	670 (7.6)	
WC (cm) M (P25~P75)	78.0 (72.0~85.0)	80.0 (74.0~87.0)	77.0 (71.0~84.0)	<0.001
abdominal overweight *N* (%)	4063 (27.2)	1500 (24.5)	2563 (29.0)	<0.001
abdominal obesity *N* (%)	1523 (10.2)	524 (8.6)	999 (11.3)	
BFP M (P25~P75)	27.0 (22.0~32.3)	23.1 (19.0~27.0)	30.4 (25.0~34.6)	<0.001
< 10% for M,< 20% for F	699 (4.7)	207 (3.4)	492 (5.6)	<0.001
10–19% for M, 20–29% for F	5264 (35.3)	1570 (25.8)	3694 (41.9)	
20–24% for M, 30–34% for F	4581 (30.7)	1999 (32.8)	2582 (29.3)	
≥ 25% for M, ≥ 35% for F	4359 (29.3)	2316 (38.0)	2043 (23.2)	
VAI M (P25~P75)	7.0 (4.0~9.0)	8.0 (5.0~11.0)	6.0 (4.0~8.0)	<0.001
1–9	10997 (75.9)	3760 (63.4)	7237 (84.5)	<0.001
10–14	2546 (17.6)	1480 (25.0)	1066 (12.5)	
15–30	945 (6.5)	687 (11.6)	258 (3.0)	

Continuous variables were presented as median (IQR) as appropriate and compared by using Mann-Whitney U test. Categorical variables were expressed as percentages and analyzed by χ^2^ test. Variables are shown as M (P25~P75) or percent; a, χ^2^ test; b, Mann-Whitney U test. BMI = body mass index, WC = waist circumference, BFP = body fat percentage, VAI = visceral adipose index.

### Prevalence of overweight/obesity and abdominal obesity in urban and rural areas

**Fig 1:**
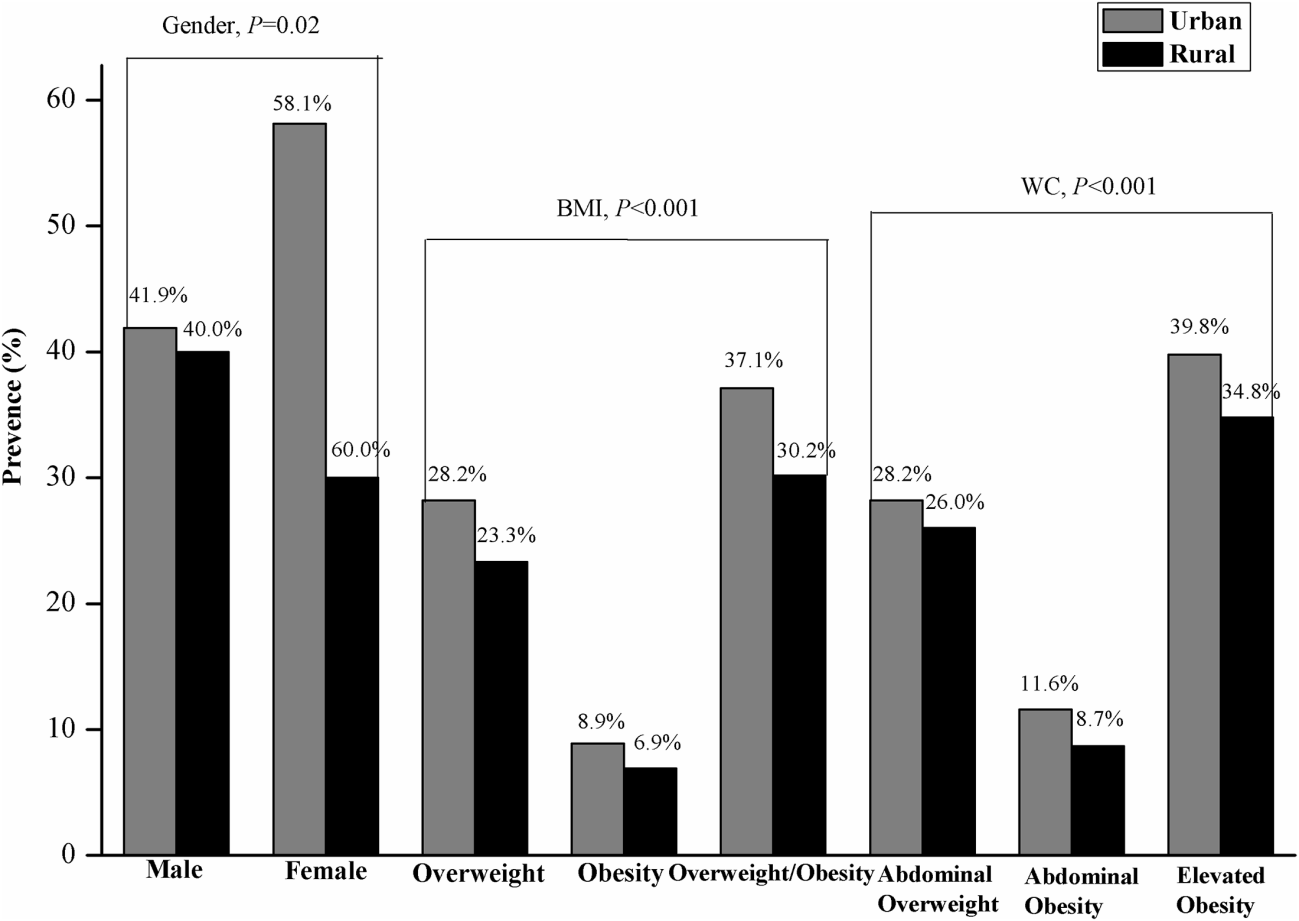
Prevalence of overweight/obesity and elevated WC in urban and rural areas. Note: BMI = body mass index, WC = waist circumference.

Fig 1 describes the prevalence of overweight/obesity and abdominal obesity in urban and rural areas. The prevalences of overweight and obesity in urban areas were 28.2% and 8.9%, respectively, and a significant urban-rural difference was observed. To our surprise, the prevalence of overweight/obesity was 37.1% in urban areas and 30.2% in rural areas, and this difference was significant (*P* < 0.001). The prevalence of abdominal obesity was significantly higher in urban areas (11.6%) than in rural areas (8.7%, *P* < 0.001). The prevalence of abdominal overweight was 28.2% in urban areas and 26.0% in rural areas. Moreover, the prevalence of elevated WC was 39.8% in urban areas and 34.8% in rural areas. The prevalence of abdominal obesity was more than that of obesity in both urban and rural areas (Fig 1).

### Prevalence of overweight/obesity and abdominal obesity stratified by sex and age

As shown in Table 2, at 15–24 years old, 16.8% of males and 7.5% of females were overweight/obesity. A significant increase in the prevalence of overweight, obesity, and abdominal obesity was seen with increasing age in both genders. Males reached their highest prevalence of obesity at 45–54 years of age, whereas females had the highest obesity prevalence at 55–64 years. The prevalences of overweight, obesity, and abdominal obesity in males were higher than those in females under 55 years of age, but in contrary, these rates were higher in females than males above 55 years of age.

**Table 2:** Prevalence of overweight/obesity, and abdominal obesity stratified by sex and age.

Age (years)	15–24	25–34	35–44	45–54	55–64	65–74	75–97	*p*–value
Male, N (%)	560 (9.1)	559 (9.1)	818 (13.4)	1032 (16.8)	1184 (19.3)	1185 (19.4)	789 (12.9)	
BMI (kg/m^2^)	20.5	22.6	23.7	23.7	22.9	22.4	21.1	
M (P25~P75)	(18.7~22.8)	(20.3~24.7)	(21.3~26.2)	(21.6~26.2)	(20.7~25.4)	(20.3~24.6)	(19.1~23.5)	<0.001
Overweight *N* (%)	66 (11.8)	132 (23.6)	272 (33.3)	356 (34.5)	344 (29.1)	297 (25.1)	122 (15.5)	<0.001
Obesity N (%)	28 (5.0)	46 (8.2)	102 (12.5)	134 (13.0)	101 (8.5)	65 (5.5)	40 (5.1)	<0.001
Overweight/Obesity N (%)	94 (16.8)	178 (31.8)	374 (45.7)	490 (47.5)	445 (37.6)	362 (30.5)	162 (20.5)	<0.001
WC (cm)	72	80.0	82.2	83.0	81.5	80.0	78.0	
M (P25~P75)	(67.7~78.0)	(74.0~85.0)	(76.6~89.0)	(76.2~90.1)	(75.0~89.0)	(74.0~86.3)	(72.0~84.0)	<0.001
abdominal overweight N (%)	38 (6.8)	121 (21.6)	261 (31.9)	329 (31.9)	331 (28.0)	290 (24.5)	130 (16.5)	<0.001
abdominal obesity N (%)	17 (3.0)	34 (6.1)	85 (10.4)	130 (12.6)	129 (10.9)	80 (6.8)	49 (6.2)	<0.001
elevated WC N (%)	55 (9.8)	155 (27.7)	346 (42.3)	459 (44.5)	460 (38.9)	370 (31.2)	179 (22.7)	<0.001
Female, N (%)	750 (8.5)	739 (8.4)	1223 (13.8)	1828 (20.7)	1834 (20.8)	1453 (16.4)	1010 (11.4)	
BMI (kg/m2)	19.7	21.3	22.4	23.1	23.6	22.8	21.5	<0.001
M (P25~P75)	(18.4~21.6)	(19.6~23.4)	(20.6~24.6)	(21.3~25.4)	(21.5~25.8)	(20.4~25.2)	(19.3~24.2)	
Overweight N (%)	47 (6.3)	115 (15.6)	309 (25.3)	574 (31.4)	624 (34.0)	403 (27.7)	201 (19.9)	<0.001
Obesity N (%)	9 (1.2)	26 (3.5)	79 (6.5)	163 (8.9)	198 (10.8)	125 (8.6)	70 (6.9)	<0.001
Overweight/Obesity N (%)	56 (7.5)	141 (19.1)	388 (31.7)	737 (40.3)	822 (44.8)	528 (36.3)	271 (26.8)	<0.001
WC (cm)	67	73.0	75.5	78.2	80.0	79.0	78.0	<0.001
M (P25~P75)	(63.6~72.0)	(68.5~79.0)	(71.0~81.0)	(73.0~84.0)	(74.0~87.0)	(73.0~86.0)	(71.0~85.0)	
abdominal overweight N (%)	45 (6.0)	121 (16.4)	319 (26.0)	634 (34.7)	690 (37.6)	472 (32.5)	282 (27.9)	<0.001
abdominal obesity N (%)	10 (1.3)	43 (5.8)	74 (6.1)	206 (11.3)	290 (15.8)	231 (15.9)	145 (14.4)	<0.001
elevated WC N (%)	55 (7.3)	164 (22.2)	393 (32.1)	840 (46.0)	980 (53.4)	703 (48.4)	427 (42.3)	<0.001

BMI = body mass index, WC = waist circumference,

### The associations between BMI, WC, VAI and BFP

**Fig 2:**
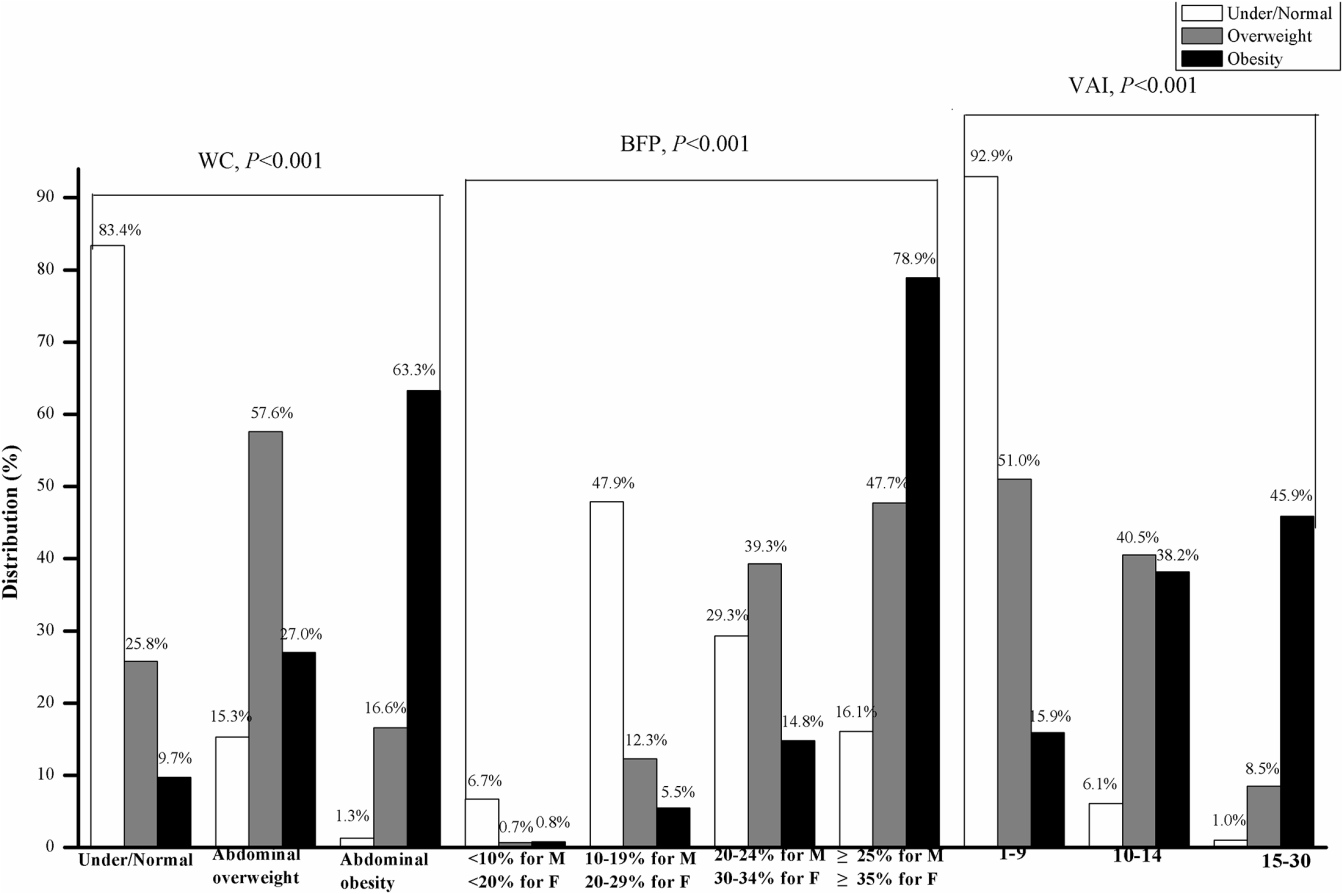
The distribution of BMI in different WC, VAI and BFP. Note: BMI = body mass index, WC = waist circumference, BFP = body fat percentage, VAI = visceral adipose index.

Fig 2 describes the associations between BMI, WC, VAI and BFP. Overall, with increasing BMI, the WC, VAI and BFP also increased significantly (*P* < 0.001). Among obese participants, 63.3% had abdominal obesity and 27.7% had abdominal overweight. Among participants with underweight/normal BMI, 1.3% still had abdominal obesity, 16.1% had high BFP and 1.0% had high VAI. Likewise, among obese participants, 9.7% had under/normal WC, 0.8% had normal BFP and 15.9% had normal VAI. Compared with normal BFP participants, those with high BFP showed the highest prevalence of overweight and obesity, which presented an ascending trend as BFP increased (*P* < 0.001). Likewise, with increasing VAI, the prevalence of obesity increased (*P* < 0.001), however, the prevalence of overweight declined (*P* < 0.001) (Fig 2). Table 3 describes the partial correlations between the indices of body fat. Pearson correlation analysis was carried out to analyze correlations between any 2 indicators of body fat. The analysis suggested that WC, BFP, VAI and BMI all had a significant correlation. The Pearson correlation coefficients between WC and BMI, VAI, and BFP were 0.711, 0.686 and 0.316, respectively (all *P* < 0.001). Additionally, the Pearson correlation coefficients between BMI and VAI and BFP were 0.673 and 0.357 (all *P* < 0.001). In addition, the Pearson correlation coefficient between BFP and VAI was 0.260 (*P* < 0.001). After controlling the confounding factors of age, sex, smoking and drinking alcohol, the partial correlation matrix showed that there was still a very high significant positive correlation between WC, VAI and BMI. The partial correlation coefficients between WC and VAI and BMI were 0.666 and 0.721, and the partial correlation coefficient between VAI and BMI was 0.700.

**Table 3:** The correlation matrix analysis of body fat evaluation indicators.

Evaluation indicator	WC	BFP	VAI	BMI
WC	1.000			
BFP	0.385*	1.000		
VAI	0.666*	0.367*	1.000	
BMI	0.721*	0.387*	0.700*	1.000

BMI = body mass index, WC = waist circumference, BFP = body fat percentage, VAI = visceral adipose index.

* *P*< 0.001; controlling the confounding of age, sex and smoking and drinking alcohol

### Risk factors for overweight/obesity and abdominal obesity

A multivariate logistic regression analysis was performed using SPSS to assess the significant determinants of overweight/obesity (BMI ≥ 24 kg/m^2^ vs BMI < 24 kg/m^2^) and abdominal obesity (yes vs no). The results showed that the female gender was significantly associated with overweight/obesity [odds ratio (OR) = 2.757, *P* < 0.001], especially abdominal obesity (OR = 6.253, *P* < 0.001). Urban residents were associated with overweight/obesity (OR = 1.221, *P* < 0.001), not including abdominal obesity. Compared with 15–24 year-old participants, people aged 25–34 years, 35–44 years, 45–54 years and 55–64 years had a greater correlation with developing overweight/obesity (OR = 2.040, 2.894, 3.031, and 2.162, respectively). Participants aged 25–35 and 45–54 years were more likely to be classified with abdominal obesity compared with those aged 15–24 years old (OR = 2.752 and 2.063, respectively). It seemed that age was not significantly associated with abdominal obesity. VAI and BFP were associated with significantly higher ORs for overweight/obesity and abdominal obesity. Compared with a thin BFP, high BFP was the factor most associated with overweight/obesity and abdominal obesity (OR = 23.902 and 14.796, respectively). Compared with standard VAI values, a slightly high VAI was the factor most associated with overweight/obesity (OR = 19.048), while a high VAI was the factor most associated with abdominal obesity (OR = 30.999). Smoking and drinking were not associated with overweight/obesity or abdominal obesity (all *P* > 0.05, Table 4).

**Table 4:** Factors associated with overweight/obesity and abdominal obesity.

Variables	Overweight/obesity (n = 5048)		Abdominal obesity (n = 1523)	
	OR (95% CI)	*p*-value	OR (95% CI)	*p*-value
Gender(Female /Male)	2.757 (1.659~4.579)	<0.001	6.253 (2.641~14.801)	<0.001
Location(Urban/ Rural)	1.221 (1.086~1.374)	0.001	1.022 (0.864~1.209)	0.801
Smoking (Yes/No)	0.985 (0.644~1.508)	0.946	1.366 (0.817~2.284)	0.235
Drinking (Yes/No)	1.038 (0.876~1.231)	0.665	1.211 (0.954~1.536)	0.115
Age (years)				
15–24	1.000		1.000	
25–34	2.040 (1.404~2.964)	<0.001	2.752 (1.327~5.706)	0.007
35–44	2.894 (2.054~4.078)	<0.001	1.313 (0.648~2.658)	0.450
45–54	3.031 (2.159~4.256)	<0.001	2.063 (1.046~4.069)	0.037
55–64	2.162 (1.488~3.142)	<0.001	1.774 (0.875~3.598)	0.112
65–74	1.263 (0.859~1.859)	0.234	1.701 (0.833~3.472)	0.145
75–97	0.636 (0.423~.0955)	0.029	1.559 (0.755~3.219)	0.230
BFP				
< 10% for M,< 20% for F	1.000		1.000	
10–19% for M, 20–29% for F	1.927 (1.257~2.954)	0.003	1.337 (0.561~3.186)	0.512
20–24% for M, 30–34% for F	11.337 (7.405~17.357)	<0.001	7.321 (3.127~17.139)	<0.001
≥ 25%for M, ≥ 35% for F	23.902 (15.447~36.985)	<0.001	14.796 (6.307~34.711)	<0.001
VAI				
1–9	1.000		1.000	
10–14	19.048 (15.092~24.040)	<0.001	7.832 (6.478~9.469)	<0.001
15–30	10.568 (7.392~15.108)	<0.001	30.999 (22.363~42.971)	<0.001

Overweight/obesity = body mass index (BMI) ≥ 24kg/m^2^, Abdominal obesity = waist circumference (WC) ≥ 95 cm in males and ≥ 90 cm in females, BFP = body fat percentage, VAI = visceral adipose index. Sex, area, age, smoking, drinking, BFP, and VAI were incorporated into the equation.

## Discussion

### Overweight and obesity

Excess body weight has demonstrated significant associations with CVD [18]. Two-thirds of the adult population in the United States and at least half the populations of many other developed countries are currently overweight or obese [19–21]. The prevalence of overweight and obesity in China has increased rapidly in the past decades. Between 1992 and 2002, the prevalence of overweight and obesity among adults increased by 39.0% and 97.2%, respectively [22]. By 2009, the prevalence of obesity among Chinese adults was 10.7% [5]. The overall prevalence of adult obesity varies not only by regions, but also by ethnic groups. This study showed that the overall prevalence of overweight/obesity among adults in Jiangxi Province, China was 33.7% (25.8% overweight and 7.9% obesity). Approximately one-third of the participants were either overweight or obesity, which is a relatively low prevalence compared to the prevalence in other regions of China and other developed countries [23–25]. This phenomenon may due to the cause of demographics distribution and environmental factors. Climate, geography and lifestyle can vary with the regions, and Jiangxi province is one of the less developed regions in China, based on the economic development levels.

This study reported that males had a slightly higher risk for obesity than females and there was no significant gender difference. Interestingly, in a multivariate analysis, the female gender was associated with the risk of overweight/obesity (OR = 2.757). In contrast to our findings, other studies have revealed that males tend to be overweight/obese [5,13,22,23]. Possible reasons for this discrepancy may be the differences in lifestyle and sociodemographic variables, as well as other genetic or behavioral factors. Furthermore, our study found a strong association between overweight/obesity and age, with the prevalence of overweight/obesity increasing as age increased in both genders. The prevalence of overweight/obesity in males was higher than that in females before 55 years old, but these rates were higher in females than males above 55 years of age. This finding concurred with some studies [23,26]. Apart from age, the rapid hormone changes during the menopausal transition may also contribute to the BMI and fat distribution changes in middle-aged women [27].

Along with economic growth and the urbanization of lifestyle in China, the prevalence of overweight/obesity among both urban and rural residents has been on the rise and the urban-rural disparity is narrowing. A recent study indicated that the prevalence of overweight and obesity increased from 36.1% to 40.1% in urban areas and from 26.7% to 38.3% in rural areas during 2000–2009 [4]. In agreement with other studies worldwide [2,4,22,24], the study also indicated that living in urban areas led to an increased correlation with overweight/obesity (OR = 1.221). This may be because urban residents mainly engage in mental activities, and expend less energy in daily life, while rural residents mostly engage in physical activities.

### Abdominal obesity

The prevalence of abdominal obesity (defined as WC ≥ 90 cm for males and ≥ 80 cm for females) among Chinese adults was 37.4% (27.8% in males and 45.9% in females) according to the China Health and Nutrition Surveys in 2009 [5]. This study reported that the prevalence of abdominal obesity (defined as WC ≥ 95 cm for males and ≥ 90 cm for females) was 10.2% (8.6% for males and 11.3% for females), which showed a trend towards a lower prevalence of abdominal obesity compared to those in other studies [28]. There may be two aspects related to this difference in prevalence. On the one hand, the values of the WC cut-off reference standards were different. On the other hand, people's health consciousness has improved. Consistent with previous studies [28,29], our findings showed that abdominal obesity is more prevalent in females than in males (OR = 6.253). Moreover, our survey indicated that the prevalence of abdominal obesity in both genders peaked in the middle and older age groups. The rapid changes in hormones, and physical activity levels may be responsible for this difference. Previous studies reported that living in urban areas would put the residents at a higher risk of developing abdominal obesity [25]. Our study also reported that the prevalence of abdominal obesity in urban areas was significantly greater than that in rural areas, but no association was found, which could be due to the difference in WC cut-off reference standards.

Those who smoke cigarettes are known to be more likely to be overweight/obese and to exhibit abdominal obesity [30,31]. However, whether alcohol consumption increases obesity is controversial [32,33]. Many studies have also reported that alcohol consumption does not necessarily lead to weight gain [32]. A prospective cohort study showed a reduction in the risk of overweight/obesity among females who consumed a little to moderate amount of alcohol compared to nondrinkers [33]. However, this study showed that there were no associations between cigarette smoking or alcohol consumption and overweight/obesity or abdominal obesity. This may be because more females than males were included in our study and because the proportion of current smokers and current drinkers was low. Moreover, the standards of smoking and drinking were not the same.

### Associations between BMI, WC, VAI and BFP

Obesity, especially abdominal obesity, is associated with cardiovascular disease. Among the indices of body fat, which can be used as the best marker to indirectly reflect cardiometabolic risk is still controversial [7–12]. At present, BMI is still considered a satisfactory predictor of the percentage of body fat in both males and females. However, our study showed that there were still a small number of participants with an underweight/normal BMI had abdominal obesity, high BFP and high VAI. Moreover, among obese participants, there was a small number who had low/normal WC, normal BFP and normal VAI. Therefore, it seems to be unreasonable that we should continue to use BMI as a cardiovascular risk factor. Further studies are needed to explore which indicators of body fat can be used as the best marker for indirectly reflecting cardiometabolic risk. Our study also presented a very high, positive correlation between WC, VAI and BMI, which was consistent with other studies [12,34]. The measure of VAI included a sex-specific model of adipose distribution based on the linear relationship between WC and BMI within each sex, and this model corrected for fat function by including triglyceride and high-density lipoprotein (HDL)-cholesterol concentrations into the equation [34]. Hence, VAI, among the most common indices of adiposity assessment, may be the most accurate indicator of visceral adipose function. However, there remains a deficit in prospective studies that can attribute a prognostic role to VAI in predicting cardiovascular risk.

This study has several limitations. First, the participants were recruited from Jiangxi Province of China; therefore the conclusion cannot represent the situation in other regions of China. Second, some variables, including education level, detailed dietary habits, family income and physical activity, were not included. Third, our study did not include chronic diseases, such as diabetes and hypertension, limiting our analyses of which indices of body fat can be used as the best marker to indirectly reflect cardiometabolic risk. The correlation between body fat indices and cardiometabolic risk requires further investigation.

In conclusion, this study described the epidemiology of obesity and abdominal obesity among adults in Jiangxi Province of China and evaluated the association between BMI, WC, VAI and BFP. The prevalence of obesity (7.9%) and abdominal obesity (10.2%) was relative low. Factors significantly associated with an increased risk of overweight/obesity and abdominal obesity were female gender, high BFP and high VAI. Living in an urban area and older age correlated with overweight/obesity. Additionally, there was a very high, significant, positive correlation between WC, BMI and VAI. Hence, further studies are needed to explore these indicators of body fat to determine which could be used as the best marker to indirectly reflect cardiometabolic risk.

## Supporting information

S1 Table Minimal data set. (XLSX)

S1 File STROBE checklist. (DOCX)
